# Pressure mediated hypertrophy and mechanical stretch up-regulate expression of the long form of leptin receptor (ob-Rb) in rat cardiac myocytes

**DOI:** 10.1186/1471-2121-13-37

**Published:** 2012-12-27

**Authors:** Hiroki Matsui, Tomoyuki Yokoyama, Chie Tanaka, Hiroaki Sunaga, Norimichi Koitabashi, Takako Takizawa, Masashi Arai, Masahiko Kurabayashi

**Affiliations:** 1 Department of Laboratory Sciences, Gunma University Graduate School of Health Sciences, 3-39-22, Showa-machi, Maebashi 371-8514, Japan; 2 Department of Medicine and Biological Sciences, Gunma University Graduate School of Medicine, 3-39-22, Showa-machi, Maebashi, 371-8514, Japan

**Keywords:** Obesity, Cardiac hypertrophy, Pressure overload

## Abstract

**Background:**

Hyperleptinemia is known to participate in cardiac hypertrophy and hypertension, but the relationship between pressure overload and leptin is poorly understood. We therefore examined the expression of leptin (ob) and the leptin receptor (ob-R) in the pressure-overloaded rat heart. We also examined gene expressions in culture cardiac myocytes to clarify which hypertension-related stimulus induces these genes.

**Results:**

Pressure overload was produced by ligation of the rat abdominal aorta, and ob and ob-R isoform mRNAs were measured using a real-time polymerase chain reaction (PCR). We also measured these gene expressions in neonatal rat cardiac myocytes treated with angiotensin II (ANGII), endothelin-1 (ET-1), or cyclic mechanical stretch. Leptin and the long form of the leptin receptor (ob-Rb) gene were significantly increased 4 weeks after banding, but expression of the short form of the leptin receptor (ob-Ra) was unchanged. ob-Rb protein expression was also detected by immunohistochemistry in hypertrophied cardiac myocytes after banding. Meanwhile, plasma leptin concentrations were not different between the control and banding groups. In cultured myocytes, ANGII and ET-1 increased only ob mRNA expression. However, mechanical stretch activated both ob and ob-Rb mRNA expression in a time-dependent manner, but ob-Ra mRNA was unchanged by any stress.

**Conclusions:**

We first demonstrated that both pressure mediated hypertrophy and mechanical stretch up-regulate ob-Rb gene expression in heart and cardiac myocytes, which are thought to be important for leptin action in cardiac myocytes. These results suggest a new local mechanism by which leptin affects cardiac remodeling in pressure-overloaded hearts.

## Background

Leptin, the product of the ob gene [1], was discovered originally as an adipocyte-derived cytokine that acts in the hypothalamus to regulate appetite and energy expenditure [2]. In spite of its anti-obese effects, serum leptin concentrations are correlated strongly with body mass index, and obesity is associated with hyperleptinemia [3,4]. Leptin is also known to have pleiotropic effects on many peripheral tissues [5]. In the cardiovascular system, hyperleptinemia has been linked to left ventricular mass in severe obese patients [6], elevated serum leptin concentrations are found in patients with congestive heart failure [7], and leptin has been identified as a risk factor for myocardial infarction [8]. Experimental data from animal studies and cultured myocytes indicate that leptin directly induces hypertrophy in cardiac myocytes [9-12]. However, another experimental study showed that leptin did not induce changes in size of rat neonatal cardiac myocytes [13]. Thus, the cardiac effects of leptin are not completely understood.

Myocardial hypertrophy is induced by various stimuli in vivo, such as pressure or volume overload [14,15]. Thus, an investigation of molecular mechanisms inducing cardiac hypertrophy in the setting of pressure overload is very important in preventing the progression of myocardial remodeling. With respect to leptin and myocardial remodeling, serum leptin concentrations are significantly associated with myocardial wall thickness in hypertensive men [16]. However, the relationship between pressure overload and leptin in cardiac myocytes has not been determined.

Moreover, leptin synthesis occurs in a variety of non-adipose cells and leptin receptors are distributed in many other tissues [17,18]. At least six alternatively spliced isoforms of the leptin receptor (ob-Ra to ob-Rf) have been identified in mice [19]. These include one long receptor protein (ob-Rb) and five shorter receptor proteins (ob-Ra, -Rc, -Rd, -Re, -Rf). The long form of the leptin receptor (ob-Rb) is similar to gp130, and acts primarily through activation of the Janus kinase (JAK) family of cytoplasmic tyrosine kinases, which in turn activate transcription factors of the signal transduction and activation of transcription (STAT) family [20].

We previously demonstrated that ischemia/reperfusion induces leptin and leptin receptor mRNA and protein expression in rat hearts, and treatment with an anti-leptin antibody prevents the increase of tumor necrosis factor-α (TNF-α) and interleukin-1β mRNA expression in ischemic hearts [21]. These studies suggest that not only increased serum leptin concentrations, but also local leptin production and up-regulation of leptin receptors may play an important role in leptin action in heart disease.

In this study, we examined induction of leptin and leptin receptor mRNA and protein expression in rat hearts after pressure overload. Furthermore, to clarify which hypertension-related stress activates leptin and leptin receptor expression, we measured leptin and leptin receptor mRNA expression in isolated ventricular myocytes stimulated with angiotensin-II (ANGII), endothelin-1 (ET-1), or mechanical stretch.

## Methods

### Animals and treatments

Thirty-five adult male Wistar rats (210 to 350 g) were submitted to experiments, whereas 18 animals were used as control groups (2 weeks; n=11, 4 weeks; n=7). In the pressure-overloaded groups, invasive hemodynamic measurements were performed 2 weeks (n=9) and 4 weeks (n=8) after banding. Cardiac hypertrophy was produced using techniques as previously described [22]. Briefly, rats were anesthetized with sodium pentobarbital (50 mg/kg, i.p.), and the suprarenal area of the descending aorta was isolated through a midline laparotomy. Then, abdominal aortic constriction surgery was performed by placing a constricting silver clip (21-gauge, inside diameter: 0.80 mm, or 22-gauge, inside diameter: 0.90 mm) around the abdominal aorta between the renal and superior mesenteric arteries. Sham-operated controls underwent the same procedure except for the placement of the aortic clip. All animals were housed according to institutional guidelines for 4 weeks in climate controlled metabolic cages with a 12-hr light/12-hr dark cycle, and food and water provided ad libitum. All experiments using these rats were approved by and performed according to the guidelines of the Animal Ethics Committee of Gunma University, Maebashi, Japan (*Permit Number*: 50153). Animals were allowed to recover for 2 to 4 weeks, and body weight averaged 309 g at 2 weeks and 324 g at 4 weeks in control rats, and 272 g at 2 weeks and 332 g at 4 weeks after banding.

### Hemodynamic measurements and echocardiographic evaluation

Pre-surgical rat blood pressure was measured using the tail-cuff technique (LE 5001 Pressure Meter; Letica SA, Barcelona, Spain). Rats were fasted for 16 hr prior to invasive hemodynamic measurements and blood sampling. Measurements of ascending aortic pressure and heart rate were measured in an unrestricted, conscious state through a heparinized indwelling polyethylene catheter that was introduced into the left carotid artery 1 day before measurement. Data were analyzed using Power Lab and Chart v5.0.1 (AD Instrument: Castle Hill, Australia). Blood was collected after hemodynamic measurements and blood plasma was separated immediately by centrifuging at 1500 g for 20 min at 4°C.

The degree of cardiac hypertrophy and left ventricular function was measured by echocardiography (EUB6000, Hitachi, Tokyo, Japan) using a 10-MHz probe. Before surgery and 14 or 28 days after surgery, rats were anesthetized with ketamine (50 mg/kg) and xylamine (10 mg/kg) injected intraperitoneally and subjected to echocardiographic study. LV mass, LV ejection fraction, and the ratio of the early to late filling wave (E/A) of the transmitral pulse-wave Doppler velocity were measured as described previously.

### Tissue preparation and total RNA extraction

Following induction of deep anesthesia, rats were killed and the hearts were excised immediately. The atria and right ventricle were removed, and the left ventricle was weighed. The degree of hypertrophy was calculated in each constricted and sham-operated rat using the ratio of left-ventricular weight (LVW) to body weight (BW). After their removal, left ventricles were frozen rapidly in liquid nitrogen and stored at −70°C until real-time quantitative RT-PCR analysis was performed. In addition, tissue blocks from hearts were fixed in 4% paraformaldehyde for 4 to 12 hr, embedded in paraffin, and sectioned at a thickness of 4-μm for immunohistochemistry. For each animal, we also measured the diameter of 10 cardiac myocytes chosen at random from left ventricles stained with hematoxylin and eosin (HE).

### Real-time quantitative RT-PCR

Total RNA was extracted from hearts using Isogen reagent (Nippon Gene, Tokyo, Japan) as described by the manufacturer’s protocol. cDNA was synthesized from 2 μg of mRNA with an RNA PCR kit (Takara, Tokyo, Japan) using an oligo-dT primer. Reaction mixtures were incubated for 30 min at 42°C, 5 min at 99°C, and 5 min at 5°C. SYBR Green quantitative PCR assays were performed using a MX3000P Multiplex Quantitative PCR System ((Agilent Technologies, Palo Alto, CA) and Brilliant SYBR Green QPCR Master Mix kit (Agilent Technologies). Primer sequences used to amplify various cDNAs are shown in Table 1. A typical PCR protocol was performed under the following conditions: 10 min at 95°C, followed by a total of 50 three-temperature cycles (95°C denaturation for 30 sec, annealing temperature for 1 min, and a 72°C extension for 1 min). Specificity of the SYBR Green amplicons was confirmed by melting point analysis and gel electrophoresis in the presence of ethidium bromide (Nippon Gene, Tokyo, Japan). Expression of the housekeeping gene GAPDH was used for normalization. All quantitative PCR assay data for ob, ob-Ra, and ob-Rb mRNA was normalized by GAPDH mRNA of the same sample, and expressed as a relative amount to the control group.

**Table 1:** Characteristics of the real-time PCR assays used in the study

**Target**	**Species**	**Sequence**		**ACC No.**
ob	Rat	forward	5^′^-CCAAAACCCTCATCAAGACC-3^′^	MN013076
		reverse	5^′^-GTCCAACTGTTGAAGAATGTCCC-3^′^	
ob-Ra	Rat	forward	5^′^-ACACTGTTAATTTCACACCAGAG-3^′^	D85557
		reverse	5^′^-AGTCATTCAAACCATAGTTTAGG-3^′^	
ob-Rb	Rat	forward	5^′^-TCTTCTGGAGCCTGAACCCATTTC-3^′^	D85558
		reverse	5^′^-TTCTCACCAGAGGTCCCTAAACT-3^′^	
GAPDH	Rat	forward	5^′^-AACGACCCCTTCATTGAC-3^′^	MN017008
		reverse	5^′^-TCCACGACATACTCAGCAC-3^′^	

Ob: leptin, ob-Ra –Rb; leptin receptor isoforms.

### Immunohistochemistry

Immunohistochemical staining was performed using a DAKO Catalyzed Signal Amplification (CSA) system (DAKO, Carpinteria, CA), according to the manufacturer’s instructions. Tissue cross sections (4-μm) including the left ventricle and interventricular septum were stained with rabbit polyclonal anti-ob-Rb (OBR13-A) antibody (Alpha Diagnostics, San Antonio, TX) at dilutions of 1:150 in Tris-buffered saline (TBS) for 15 min at room temperature. Counterstaining was done with 2% methyl green. Kidney tissue was used as a positive control, while negative controls were produced by substituting nonimmune mouse immunoglobulin (DAKO) for the primary antibody.

To determine ob-Rb protein expression semi-quantitatively, we evaluated the percentage of area staining positive relative to the total area from 12 randomly chosen fields in left ventricles using Image J photographic software (National Institutes of Health).

### Western blot analyses

Whole heart extracts were prepared using modified RIPA buffer containing 50 mM Tris–HCl (pH7.4), 150 mM NaCl, 1% Nonidet P-40, 0.25% sodium deoxycholate, 1 mM EDTA, and containing complete mini and phosSTOP solution (Roche Diagnostics, Tokyo, Japan). The mixture was rotated at 4°C for 15 min and centrifuged at 14,000 rpm for 15 min. Western blot analysis was performed according to standard procedures using the following primary antibodies: polyclonal leptin (PA1-051; Thermo Scientific, Rockford, IL), mouse monoclonal anti-glyceraldehyde-3-phosphate dehydrogenase (GAPDH) (MAB374; Millipore, Billerica, MA), rabbit monoclonal phospho-STAT3 [Tyr705] (#9145) and STAT3 (#4904) mAb (Cell Signaling, Tokyo, Japan). Antigens were revealed by Immobilon Western Chemiluminescent HRP Substrate (Millipore, Bedford, MA) after incubation with horseradish peroxidase-conjugated anti-rabbit IgG.

For quantitative analysis, western blot analysis data for phospho-STAT3 was normalized by total STAT3 protein of the same sample using Image J photographic soft ware, and the STAT3 activity of banding group was expressed as a relative amount to control group in respective time.

### Measurements of plasma leptin concentrations

Rat blood was collected from the ascending aorta and plasma concentrations of leptin were evaluated using a leptin rat ELISA system (Amersham Pharmacia Biotech, Piscataway, NJ), according to the manufacturer’s instructions. The optical densities of the ELISA samples were determined within 30 min on a microplate reader set to 450 nm (DYNEX MRX Revelation, Dynex Technologies, Chantillly, VA).

### Preparation of neonatal rat cardiac myocytes

Primary neonatal rat cardiac myocyte cultures were prepared as previously described [23]. Cardiac myocytes were plated 2.5 × 10^6^ cells in 60-mm culture dishes, and 2 × 10^5^ cells per well for mechanical stretch. Using this method, we routinely obtained cardiac myocyte-rich cultures with >95% of the cells being cardiac myocytes, as assessed by immunocytochemical staining with monoclonal antibodies against sarcomeric α-actinin (Sigma Chemical Co., St. Louis, MO).

### Mechanical stretch and humoral factor stimulation of cardiac myocytes

Cyclic mechanical stretch was performed in vitro using a Flexcell strain unit (Flexcell International, McKeesport, PA). Cardiac myocytes were cultured in six-well plates with flexible gelatin-coated silicone rubber membranes at the bottom of each well. A vacuum (~28 kPa) was applied at a frequency of 6 cycles/min (4-sec on time, 6-sec off time) to the flexible membrane from the base of the plate. The maximal percent elongation of the culture surface was 18%. Previously, we reported that this protocol was sufficient to demonstrate cellular responses in stretched myocytes [23]. Following 1 to 24 hr of mechanical stretch, cells were collected with phosphate-buffered saline (PBS; pH 7.4) with 1% EDTA.

Serum-starved (24 hr) cardiac myocytes were incubated in the presence or absence of angiotensin-II (ANGII: 1 to 10 μM) or endothelin-1 (ET-1: 0.01 to 0.1 μM). After 24 hr of incubation at 37°C, mRNA was collected from the cells.

We previously showed using the same neonatal rat cardiac myocyte cultures that 0.1 μM of ET-1 increased cell size and expression of atrial natriuretic factor m-RNA [24], and stimulation with cyclic mechanical stretch for 6 hr increased phenylalanine incorporation in cardiac myocytes 40.8% higher than that in control cells [23]. Thus, we used this in vitro model for the model of cardiac myocyte hypertrophy.

### Statistical analysis

Values are reported as the mean ± SD. To compare the value at 2 or 4 weeks after banding to the control value at each period, we used student t test for analysis. To evaluate differences between multi-groups, we used one way ANOVA. Where appropriate, post hoc multiple comparison tests were performed to evaluate differences between the control and experimental groups. A p value <0.05 was considered significant.

## Results

### Cardiac structure and function in pressure-overloaded rat hearts

**Figure 1:**
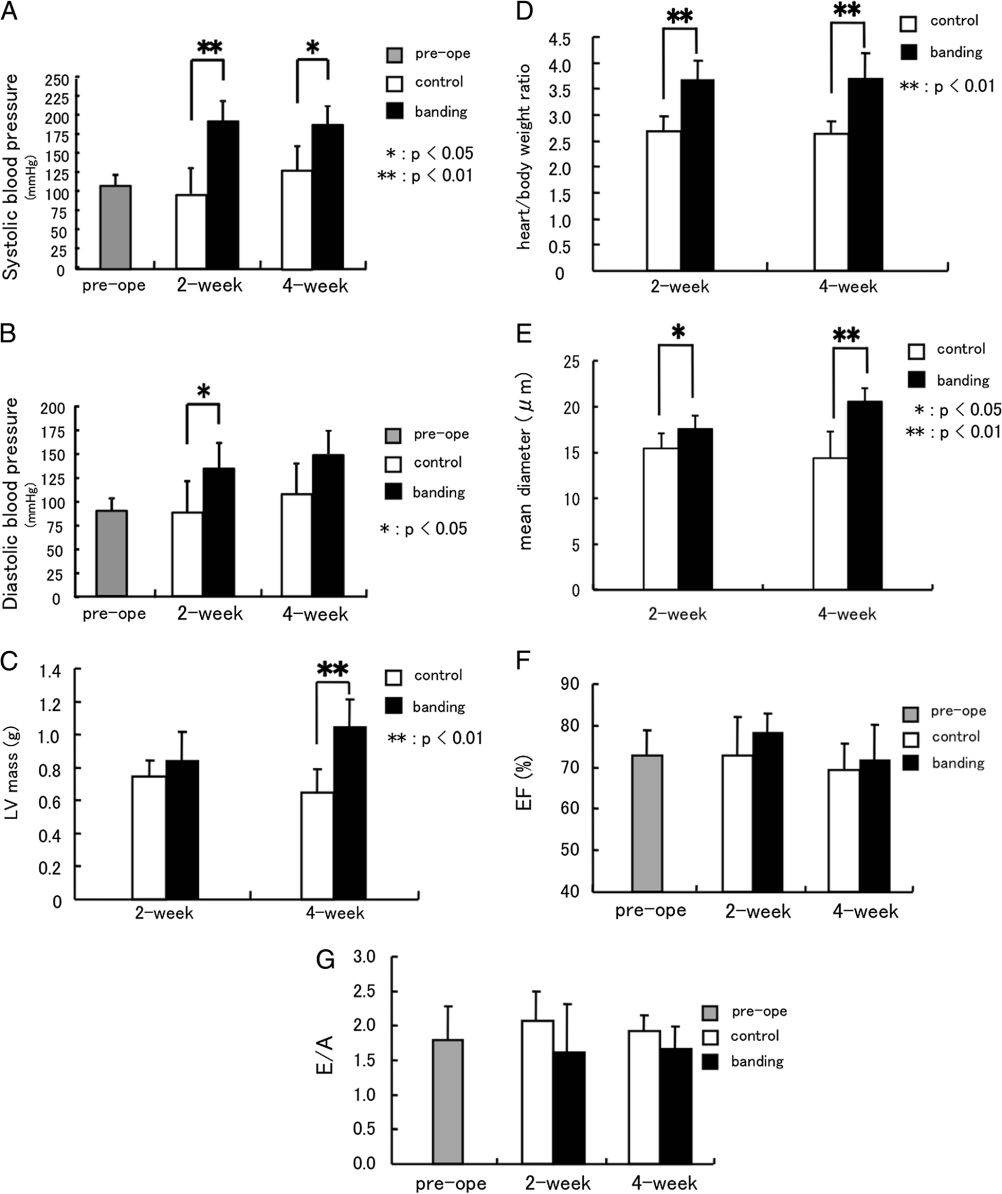
**Cardiac structure and function in pressure-overloaded rat hearts.** Blood pressure in the control group and the pressure-overloaded group (**A**), (**B**); systolic blood pressure (**A**) and diastolic blood pressure (**B**), pre: n=11, 2w: control n=6, banding n=4, 4w: control n=4, banding n=5. LV mass (**C**), heart/body weight ratio (**D**) and mean diameters of left ventricular cardiac myocytes (**E**) in the control group and the pressure-overloaded group, 2w: control n=11, banding n=9, 4w: control n=7, banding n=8. Echocardiographic evaluation of left ventricular function (**F**), (**G**). Ejection fraction (**F**) and the transmitral E/A ratio (**G**), pre: n=13, 2w: control n=5, banding n=7, 4w: control n=5, banding n=5. Values are reported as means ± SD. *p<0.05, **p<0.01 vs. control.

To assess for evidence of cardiac hypertrophy, cardiac functional parameters were carefully monitored. Systolic and diastolic pressures measured by catheter implanted into carotid artery in the pressure-overloaded group were significantly elevated over the control group at 2 weeks after banding, and systolic but not diastolic pressure was increased in the pressure-overloaded group at 4 weeks after banding (Figure 1A and B). There were no differences in presurgical BW between the two groups. Although the LVW was significantly increased in the 4 weeks pressure-overloaded group but not in the 2 weeks banding, the LVW/BW ratios in the 2 and 4 weeks pressure-overloaded groups were significantly higher than those of the respective control groups (Figure 1C and D). We also measured the diameter of cardiac myocytes in the left ventricular sections with HE staining. The mean diameters in the pressure-overloaded groups were significantly higher than those of the respective control groups (Figure 1E). Furthermore, M-mode echocardiography demonstrated concentric LV hypertrophy, while the ejection fraction was similar to that in control rats (Figure 1F). In pressure-overloaded rats, transmitral Doppler velocity showed decreased early and increased late filling velocities, resulting in a reduced E/A compared with control rats, although the difference was not significant (Figure 1G). These data indicate the induction of cardiac hypertrophy without the development of congestive heart failure in the experimental group.

### mRNA expression of ob and ob-R isoforms in pressure-overloaded hearts

**Figure 2:**
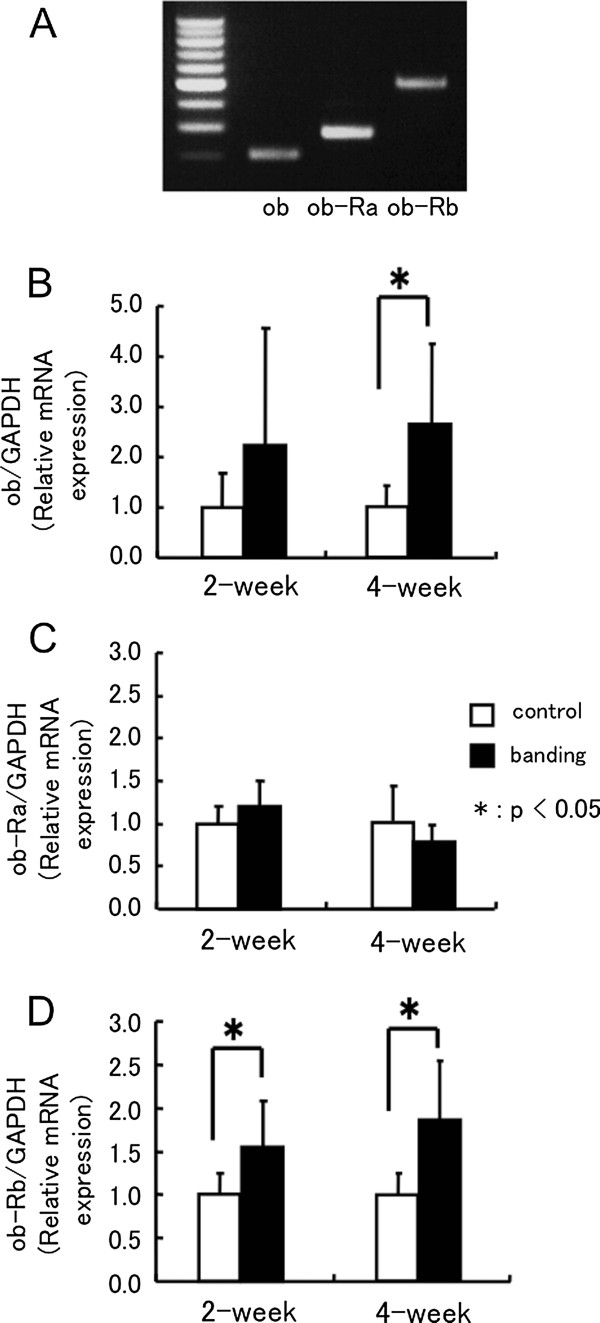
**mRNA expression of ob and ob-R isoforms in pressure-overloaded hearts.** mRNA expression of the ob and ob-R isoforms in the normal rat heart (**A**). mRNA levels of ob (**B**), ob-Ra (**C**), and ob-Rb (**D**) isoforms in pressure-overloaded rat hearts. All quantitative PCR assay data for ob, ob-Ra, and ob-Rb mRNA is normalized by GAPDH mRNA of the same sample, and expressed as fold increase compared with the mean level of control groups at 2 or 4 weeks after surgery. 2w: control n=11, banding n=9, 4w: control n=7, banding n=8. Values are reported as means ± SD. *p<0.05 vs. control.

In normal rat hearts, both ob and ob-R mRNA expression were detectable by RT-PCR (Figure 2A). Among the ob-R isoforms, mRNA for the ob-Ra isoform was expressed at a high level, whereas ob-Rb expression was detected at lower levels than ob-Ra expression. To determine the effects of pressure-overload on the expression of ob and ob-R isoforms in rat hearts, ob and ob-R mRNA expression was determined using real-time quantitative RT-PCR 2 weeks or 4 weeks after surgery. As shown in Figure 2 B and D, ob mRNA increased significantly at 4 weeks and ob-Rb mRNA increased significantly at 2 and 4 weeks after aortic banding (ob/GAPDH, 2w: 2.2±2.3 fold, 4w: 2.7±1.6 fold*, ob-Rb/GAPDH, 2w: 1.6±0.5 fold*; 4w: 1.9±0.7 fold* relative to control, *p<0.05). On the other hand, there was big individual difference in the ob gene expression every animal at 2 weeks after banding, and no significant difference was shown compared to control group. At the same time, ob-Ra mRNA expression did not change between the two groups (Figure 2C).

### Localization of ob-Rb protein expression in pressure-overloaded hearts

**Figure 3:**
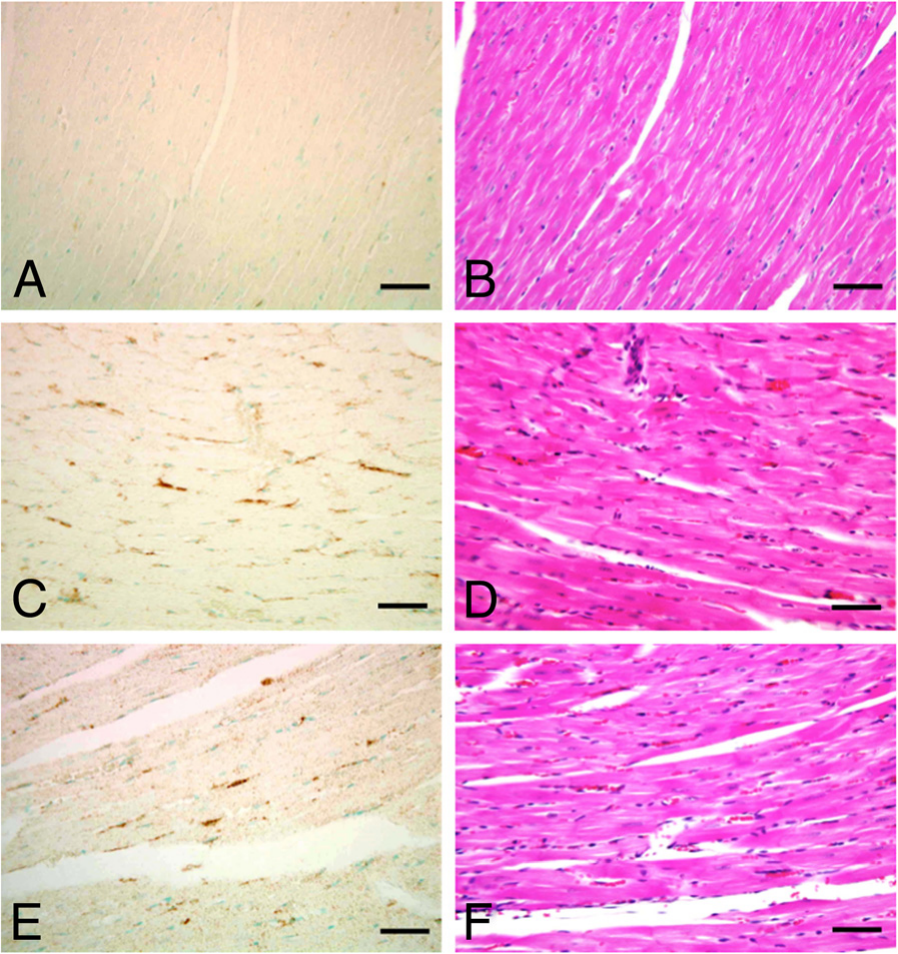
**Localization of ob-Rb protein expression in pressure-overloaded hearts.** Immunohistochemical staining for ob-Rb protein (**A**, **C**, and **E**) and corresponding HE-stained sections (**B**, **D**, and **F**) of rat myocardium after aortic constriction. (**A**), (**B**): control heart; (**C**), (**D**): 2-week pressure-overloaded rat heart, and (**E**), (**F**): 4-week pressure-overloaded rat heart. Original magnification, x400 for (A) through (**F**). Bar=0.05 mm. Counterstaining was done with 2% methyl green.

To determine the expression of ob-Rb proteins in hypertrophic hearts, we performed immunohistochemical staining in ventricular sections with anti-ob-Rb antibodies 2 and 4 weeks after aortic constriction. Although minimal ob-Rb staining was observed in the control hearts (Figure 3A), the area of ob-Rb positively staining was significantly increased in the pressure-overloaded group after 2 and 4 weeks (Figure 3C and E and Table 2). When we observed the detailed location of ob-Rb positive staining area, with HE staining as a guide (Figure 3D and F), ob-Rb was expressed in cytoplasmic membranes of hypertrophic cardiac myocytes, but not in normal cardiac myocytes or non-myocytes.

**Table 2:** Semi-quantification of ob-Rb protein expression

	**Control**	**Banding**
2-week	0.057 ± 0.094	0.181 ± 0.113*
	(n=11)	(n=9)
4-week	0.028 ± 0.021	0.103 ± 0.058*
	(n=7)	(n=8)

Values are percentage of area staining positive relative to the total area in the left ventricles (mean ± SD). *: p<0.05 vs. control.

### STAT3 activity in pressure-overloaded hearts

**Figure 4:**
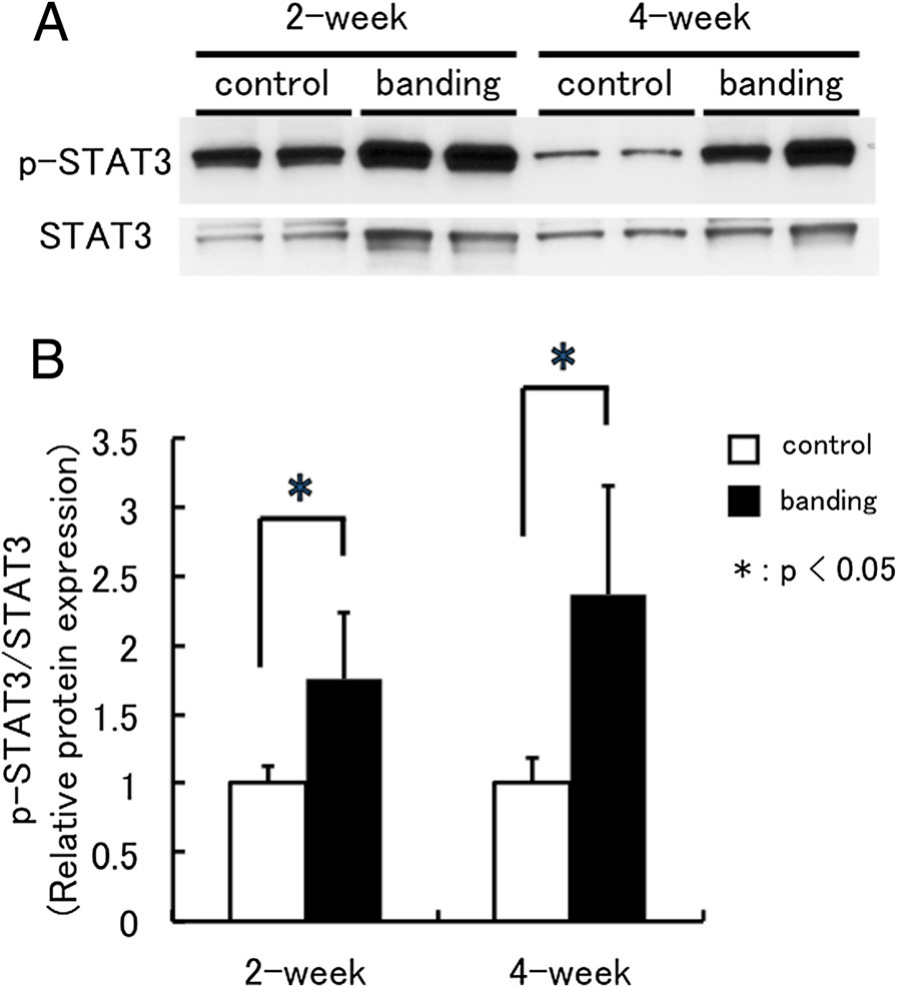
**STAT3 activity in pressure-overloaded hearts.** STAT3 activity in the control rat hearts (control) and pressure-loaded rat hearts (banding) at 2 and 4 weeks after surgery (**A**). Phosphorylated STAT3 content (p-STAT3) and total STAT3 content (STAT3) in hearts were determined with Western blot analysis. Western blot data for p-STAT3 is normalized by total STAT3 of the same sample, and expressed fold increase compared with mean level of control group (**B**). 2w: control n=3, banding n=5, 4w: control n=3, banding n=3. Values are reported as means ± SD. *p<0.05 vs. control.

As shown in Figure 4A and B, the content of phosphorylated STAT3 in control rat hearts at 2 weeks after sham operation was stronger than that at 4 weeks later and the content of phosphorylated STAT3 was increased in rat hearts after banding compared to that in control rats. The relative amount of phosphorylated STAT3 to respective control group was significantly increased in rat hearts at 2 and 4 weeks after banding. Thus pressure overload induced STAT3 activity in the heart.

### Leptin protein level in pressure-overloaded hearts

**Figure 5:**
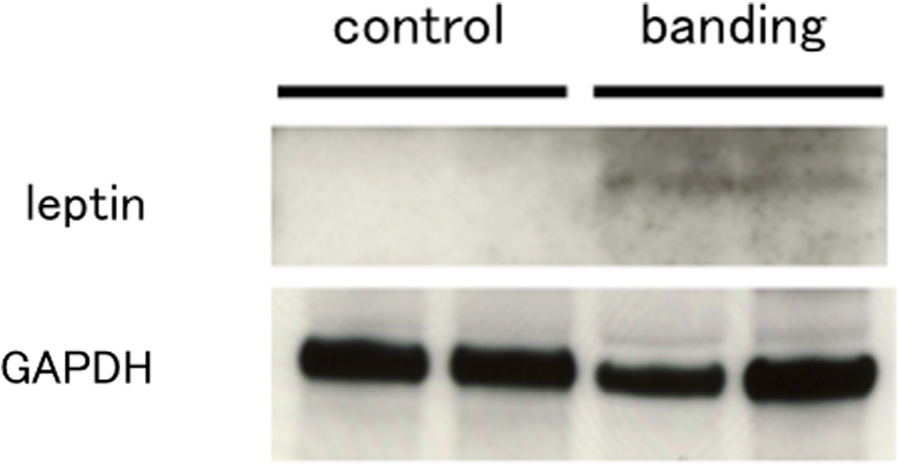
**Leptin protein level in pressure-overloaded hearts.** Leptin protein level in the control rat hearts (control) and pressure-loaded rat hearts (banding) at 4 weeks after surgery. Leptin content in hearts was determined with Western blot analysis. GAPDH was measured as an internal control. n=2 in each group.

To determine the leptin protein level in hypertrophic hearts, we performed Western blot analysis. The tissue leptin protein level in rat heart was increased in 4 weeks after banding compared to relative control (Figure 5).

### Plasma leptin concentrations in pressure-overloaded and sham-operated groups

To determine whether hypertensive stress modulates the plasma leptin concentration of pressure-overloaded rats, we performed rat leptin ELISA assays. However, we could not show differences between the control and banding groups (Table 3).

**Table 3:** Plasma leptin concentration

	**Control**	**Banding**
2-week	3.62 ± 2.11	3.40 ± 2.54
	(n=7)	(n=4)
4-week	3.89 ± 1.82	3.22 ± 1.41
	(n=4)	(n=5)

Values are mean ± SD (ng/ml).

### Effect of ANG-II, ET-1, and cyclic mechanical stretch in cardiac myocytes

**Figure 6:**
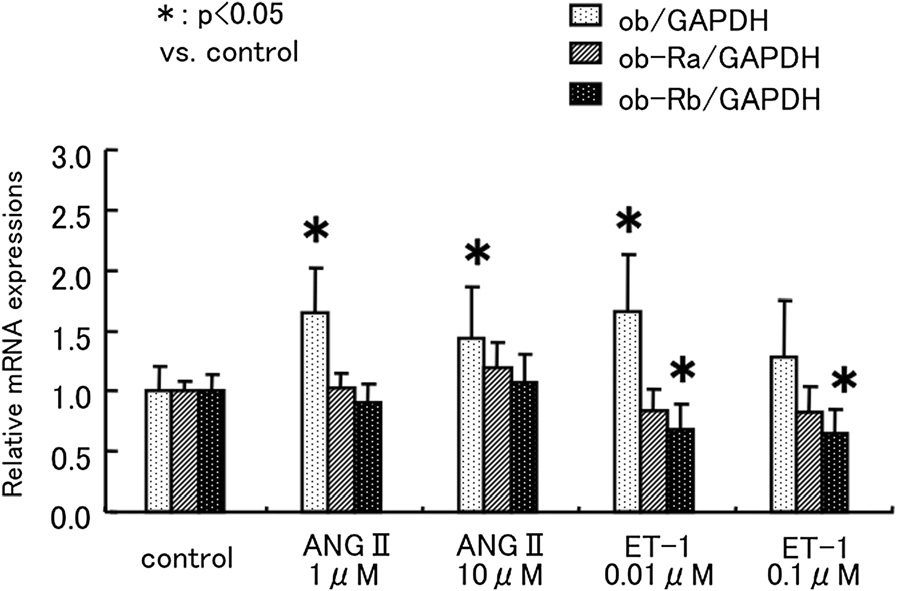
**Effect of ANG-II and ET-1 in cardiac myocytes.** Effect of neurohumoral factors on ob, ob-Ra, and ob-Rb mRNA expression in neonatal rat cardiac myocytes. Neonatal rat cardiac myocytes were treated with ANGII (1 to 10 μM), ET-1 (0.01 to 0.1 μM) for 24 h. All quantitative PCR assay data for ob, ob-Ra, and ob-Rb mRNA is normalized by GAPDH mRNA of the same sample, and expressed as fold increase compared with the mean level of control dishes. n=4 dishes in each group. Values are reported as means ± SD. *p<0.05 vs. control.

**Figure 7:**
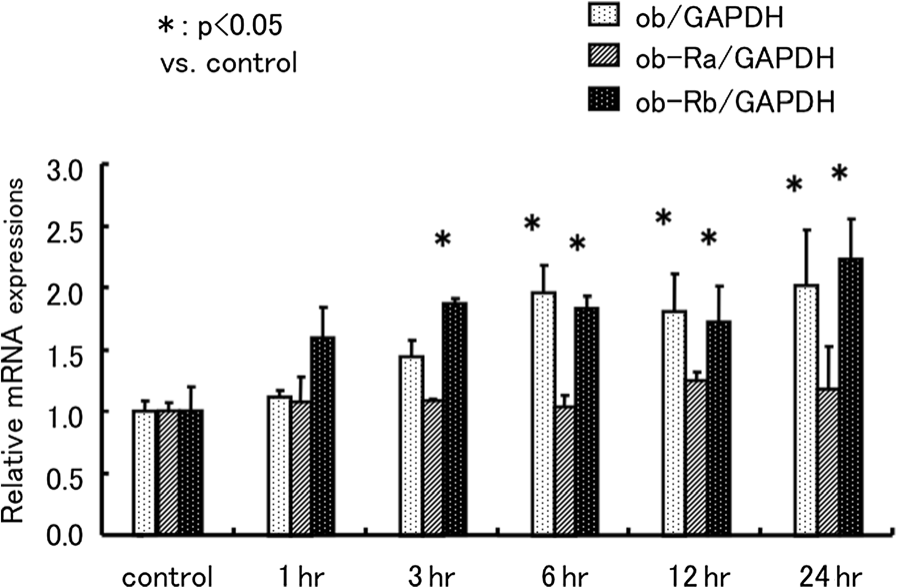
**Effect of cyclic mechanical stretch in cardiac myocytes.** Effect of cyclic mechanical stretch on ob, ob-Ra, and ob-Rb mRNA expression in neonatal rat cardiac myocytes. Neonatal rat cardiac myocytes were treated with mechanical stretch for 1 to 24 h. All quantitative PCR assay data for ob, ob-Ra, and ob-Rb mRNA is normalized by GAPDH mRNA of the same sample, and expressed as fold increase compared with the mean level of control wells. n=4 dishes in each group. Values are reported as means ± SD. *p<0.05 vs. control.

On the basis of the above observations, we concluded that ob and ob-Rb are induced by pressure-overload stress in cardiac myocytes. Therefore, additional experiments were performed using cardiac myocyte cultures to clarify which hypertensive stresses, including mechanical stretch and neurohumoral factors, increase ob and ob-Rb expression. Therefore, we examined mRNA expression in neonatal rat cardiac myocytes treated with ANGII (1 to 10 μM), ET-1 (0.01 to 0.1 μM), or cyclic mechanical stretch. ANGII and ET-1 stimulated ob mRNA expression (1 μM ANGII: 1.7 ± 0.4 fold*, 0.01 μM ET-1: 1.7 ± 0.5 fold* relative to control, *p<0.05), but ob-Ra and ob-Rb expression were unchanged by ANGII. Further ob-Rb expression was reduced, and ob-Ra expression was unchanged by ET-1 (Figure 6). In contrast, mechanical stretch induced ob and ob-Rb mRNA expression in a time-dependent manner (ob/GAPDH: 2.0 ± 0.5 fold*, ob-Rb/GAPDH: 2.2 ± 0.3 fold* relative to control, *p<0.05). However, ob-Ra was also unchanged by mechanical stretch (Figure 7).

## Discussion

With respect to leptin and cardiac hypertrophy, clinical investigations have demonstrated that fasting plasma leptin concentrations are associated with increased myocardial wall thickness independent of body weight and blood pressure in hypertensive men [16]. Many researchers have also consistently provided strong evidence that leptin induces hypertrophy in cultured neonatal rat ventricular myocytes [9-12], and the hypertrophic effects of leptin were prevented by antibodies to ob-Ra and ob-Rb [25]. The direct effects of leptin for cardiac function in which administration of leptin to adult rat ventricular myocytes attenuated cardiac contraction were also observed [26]. Although systolic and diastolic cardiac function parameters measured by M-mode echocardiography at 2 or 4 weeks after pressure overload were not decreased in our study, 2 month-lasting pressure-overloaded model showed a worsening of LV contraction [27]. Thus, 4 weeks of pressure overload can induce cardiac hypertrophy, but may be not enough to reduce cardiac function.

On the other hand, recent studies in mice demonstrated the anti-hypertrophic effect of leptin in which the blunting leptin signaling through a leptin-deficiency or leptin receptor-deficiency is associated with progressive ventricular hypertrophy in these obese mice [28,29]. In addition, a cardiac myocyte-specific reduction of ob-R expression in mice results in worse cardiac hypertrophy and function after experimental myocardial infarction [30]. With respect to pressure overload hypertrophy, left ventricular mass in leptin deficient mice subjected to transverse aortic constriction was significantly higher than in wild type mice [31]. Furthermore, a recent study shows that low serum leptin levels are associated with increased cardiovascular events and mortality in patients with stable coronary artery disease [32]. This prospective cohort study suggests the protective effect of leptin for heart disease. Thus, the role of leptin on heart disease is still controversial both in experimental and human studies. Although the direct effects of leptin on the progression of cardiac hypertrophy are still controversial and is complicated, as mentioned in above, the local induction of leptin and/or leptin receptor expression on cardiac myocytes should be important to the progression of cardiovascular diseases, even if leptin has an anti-hypertrophic effect.

Our study demonstrates that ob gene mRNA can be detected in rat hearts by real-time PCR. Furthermore, the expression of ob mRNA and the leptin protein level in the heart increased 4 weeks after aortic banding. Since circulating plasma leptin concentrations were not different between control and pressure-overloaded rats, the local production of leptin could be important for the action of leptin on cardiac myocytes. The relationship between plasma leptin concentrations and blood pressure in animal models and in humans has been investigated [33-35]. However, most of these studies found that the positive relationship between leptin and blood pressure is dependent on total fat mass [36,37]. Thus, whether high blood pressure without obesity stimulates leptin synthesis in adipocytes and/or other types of cells is not known. In this study, heart ob gene expression was increased 4 weeks after banding, but this increase was not parallel with the increase of systolic or diastolic blood pressure. Thus, some humoral factor associated with pressure overload, e.g. ANGII or ET-1, may involve the up-regulation of leptin gene.

In cultured cardiac myocytes, many stimuli induce cardiac hypertrophy, including mechanical stretch, ANGII, and ET-1. To clarify which hypertension-related stimulus induces ob and ob-R mRNA expression in cardiac myocytes, we examined their mRNA expressions in neonatal rat ventricular myocytes treated with ANGII, ET-1, or cyclic mechanical stretch. All three stimuli increased the expression of ob mRNA, but ob-Rb mRNA only increased with mechanical stretch and ob-Ra mRNA was unaffected by any stimulus. These results, together with the increased expression of ob and ob-Rb mRNA in hearts after aortic banding, strongly suggest that leptin affects cardiac myocytes during pressure overload in a local manner. Recently, similar results were reported using other types of cells in which stretch activated ob and ob-Rb expression in a cultured rat portal vein [38]. Moreover, ANGII and ET-1 promote leptin production in cultured adipocytes and neonatal rat ventricular myocytes [25,39]. Although we showed that ob mRNA expression was induced by both mechanical stretch and humoral factors, neither ANGII or ET-1 up-regulated ob-Ra and ob-Rb mRNA expression in neonatal rat cultured cardiac myocytes, Thus mechanical stretch is the most important stimulation for ob-Rb gene induction in our study. However, Rajapurohitam et al. [25] showed that both ANGII and ET-1 stimulated the gene expression of ob-Ra and ob-Rb using similar cultured cardiac myocytes. The exact reason for the discrepancy between our results and this study is not known. The different conditions of cell culture may lead to different responses to ANGII and ET-1.

The murine leptin receptor exists in six isoforms formed by alternative splicing [40]. All isoforms have the same extracellular domain, and ob-Ra, -Rc, -Rd, and -Rf contain short intracellular domains, whereas only ob-Rb has a long cytoplasmic domain [41]. De Matteris et al. [42] studied localization of leptin receptor isoforms in mouse peripheral tissues by immunohistochemistry. Their results indicate that most of the organs studied expressed ob-Ra, including the heart, and also expressed ob-Rb, although staining for ob-Rb was weaker. Using real-time PCR, at least three of the leptin receptor isoforms, including ob-Ra, ob-Rb, and ob-Re, can be detected in adult rat hearts [43]. Moreover, long and short isoforms of the leptin receptor and intracellular proteins mediating leptin signaling are expressed in human hearts based on real-time PCR and immunohistochemistry [6]. We demonstrated by the real-time PCR that both short and long forms of the leptin receptors are expressed in rat hearts as well as neonatal rat ventricular myocytes. Expression of ob-Rb was weaker than that of ob-Ra in rat hearts, in keeping with previous reports. However, ob-Rb mRNA and protein expression were significantly increased 2 to 4 weeks after aortic banding in comparison with control rats, whereas ob-Ra expression was unchanged. Unlike ob-Rb, ob-Ra is continuously expressed in hearts. We first showed that pressure overload and mechanical stretch induce ob-Rb, but not ob-Ra in cardiac myocytes both in in vivo and in vitro models. These results emphasize the role of the long form leptin receptor in leptin-associated heart disease. On the other hand, both ob-Ra and ob-Rb genes and protein expressions were increased in spontaneously hypertensive rat vascular smooth muscle cells [44]. Regulation of leptin receptor expression may be different depending on tissue or cell types.

The long form of the leptin receptor (ob-Rb) activates JAK/STAT pathways, and ob-Rb activation also may phosphorylate JAK leading to the activation of insulin receptor substrate (IRS-1) and mitogen-activated protein kinase (MAPK). The short form of the leptin receptor (ob-Ra) phosphorylates IRS-1 and consequently activates MAPK, but appears to be unable to activate the JAK/STAT pathway [5,45]. Mice lacking only the ob-Rb, but not the ob-Ra gene have an indistinguishable phenotype from all leptin receptor deficient mice [4,19]. Thus, ob-Rb and the ob-Rb activating JAK/STAT pathways are crucial for leptin action. Several experiments have shown that leptin stimulates various intracellular signaling cascades in cardiac myocytes, including JAK/STAT, extracellular signal-regulated kinase (ERK)1/2, p38 MAPK, RhoA/Rho kinase (ROCK) system, reactive oxygen species, and nitric oxide pathways (reviewed by Karmazyn et al. [46]).

Rajapurohitan et al. [9] demonstrated that the hypertrophic effects of leptin were inhibited by a p38 MAPK inhibitor, and they identified only ob-Ra mRNA in neonatal rat cardiac myocytes. In contrast to this previous study, our results demonstrate that both ob-Ra and ob-Rb mRNA are present in isolated neonatal rat ventricular myocytes as well as adult rat hearts, and mechanical stretch activates ob-Rb mRNA expression in cardiac myocytes. Although leptin mediated hypertrophy, at least in part through a p38 MAPK pathway [11,47], ob-Rb, but not ob-Ra, activates the JAK/STAT pathway, and has a prominent role in leptin signaling. Abe et al. [12] demonstrated that leptin induces cardiac myocyte elongation via the JAK/STAT pathway, but not through the MAPK pathway. A recent study using ob-Rb deficient db/db mouse showed that leptin induced inflammatory response of adipose tissue amplified through ob-Ra when ob-Rb is defective [48]. Ob-Ra may transmit leptin signal to cardiac myocytes independently from pressure over load or mechanical stretch.

Meanwhile, McGaffin et al. [30] demonstrated that intact cardiac leptin signaling in ischemic heart failure is important for the maintenance of glycolytic metabolism, involves the activation of STAT3, and attenuates adverse cardiac remodeling post myocardial infarction. Mice with cardiac myocyte-specific overexpression of STAT3 present increased capillary density and develop a concentric hypertrophy with preserved cardiac function [49]. Furthermore, mice with the gp130 gene, an up-stream component of STAT3, knocked out in cardiac-specific manner showed a decompensation in response to pressure overload [50]. Despite the induction of cardiac hypertrophy by activation of JAK/STAT signaling, STAT3 plays a key role in cardio-protection in response to numerous stress situations including pressure overload. Although we did not intend to show the direct effect of ob-Rb to the JAK/STAT pathway in this study, pressure overload induced STAT3 activity in the heart. The increased number of leptin attached to the long form leptin receptor in cardiac myocytes under pressure overload and mechanical stretch may activate STAT3 and result in the cardio-protective effect of leptin.

## Conclusions

Adipocytokines including leptin have now attracted more attention due to their distinct effects in the cardiovascular system and their predictive values for future cardiaovascular events, especially in obese subjects [51]. In this study, we first demonstrated that both pressure mediated hypertrophy and mechanical stretch up-regulate the long form of the leptin receptor gene expression in heart and cardiac myocytes, which is thought to be important for leptin action in cardiac myocytes. Although previous reports are divided on whether leptin has predominantly adverse or beneficial effects on cardiac hypertrophy, our results suggest a new local mechanism by which leptin directly affects cardiac remodeling in pressure-overloaded hearts.

## Competing interests

The authors declare that have no competing interests.

## Authors’ contributions

HM conceived of the study, participated in its design and wrote the manuscript. YT conceived of the overall study design and coordination, helped to draft the manuscript. CT carried out the in vivo studies, analysis of RNA and ELISA assays. HS carried out western blots. NK directed in vivo studies. TT directed in vivo studies. MA participated in the design of the study. MK participated in the design of the study and helped to draft the manuscript. All authors read and approved the final manuscript.
